# MLGCN: an ultra efficient graph convolutional neural model for 3D point cloud analysis

**DOI:** 10.3389/frai.2024.1439340

**Published:** 2024-09-20

**Authors:** Mohammad Khodadad, Ali Shiraee Kasmaee, Hamidreza Mahyar, Morteza Rezanejad

**Affiliations:** ^1^Department of Computational Science and Engineering, McMaster University, Hamilton, ON, Canada; ^2^Rosalind and Morris Goodman Cancer Institute, McGill University, Montreal, QC, Canada

**Keywords:** 3D point cloud, 3D shape analysis, Graph Neural Networks, efficient networks, graph KNNs

## Abstract

With the rapid advancement of 3D acquisition technologies, 3D sensors such as LiDARs, 3D scanners, and RGB-D cameras have become increasingly accessible and cost-effective. These sensors generate 3D point cloud data that require efficient algorithms for tasks such as 3D model classification and segmentation. While deep learning techniques have proven effective in these areas, existing models often rely on complex architectures, leading to high computational costs that are impractical for real-time applications like augmented reality and robotics. In this work, we propose the Multi-level Graph Convolutional Neural Network (MLGCN), an ultra-efficient model for 3D point cloud analysis. The MLGCN model utilizes shallow Graph Neural Network (GNN) blocks to extract features at various spatial locality levels, leveraging precomputed KNN graphs shared across GCN blocks. This approach significantly reduces computational overhead and memory usage, making the model well-suited for deployment on low-memory and low-CPU devices. Despite its efficiency, MLGCN achieves competitive performance in object classification and part segmentation tasks, demonstrating results comparable to state-of-the-art models while requiring up to a thousand times fewer floating-point operations and significantly less storage. The contributions of this paper include the introduction of a lightweight, multi-branch graph-based network for 3D shape analysis, the demonstration of the model's efficiency in both computation and storage, and a thorough theoretical and experimental evaluation of the model's performance. We also conduct ablation studies to assess the impact of different branches within the model, providing valuable insights into the role of specific components.

## 1 Introduction

With advances in 3D acquisition technologies, 3D sensors are becoming more accessible and cost-effective. Sensors including 3D scanners, LiDARs, and RGB-D cameras (e.g., RealSense, Kinect, and Apple depth cameras) provide a wealth of information about the shape, scale, and geometry of objects in the environment. Consequently, there has been an increasing need to develop algorithms and models for point cloud analysis, and 3D model classification and segmentation have become active areas of research in machine learning and computer vision. Deep learning techniques have proven to be highly effective for this task due to their ability to learn rich features and representations from raw data. However, most existing 3D deep learning models rely on large and complex architectures, making them computationally expensive and unsuitable for real-time applications, such as augmented reality, robotics, and autonomous driving.

Most sensors on modern 3D perception devices acquire data in the form of point clouds, and traditionally, researchers sample this data on voxel grids for 3D volumetric convolutions. However, the use of low-resolution can result in information loss, e.g., when multiple points fall within the same voxel. To preserve necessary detail in the input data, a high-resolution representation is preferable, but this can lead to an increase in computational costs and memory requirements. Whereas data acquired by sensors is often in the form of 3D point clouds, they are unordered and sparse, requiring models that are permutation agnostic and multi-scale. Whereas classical Convolutional Neural Network (CNN) models have been effective for image-based computer vision problems, they cannot be directly applied to 3D point cloud analysis.

In recent years, numerous powerful models have been proposed to analyze point clouds (Qi et al., 2017b; Qiu et al., 2021; Rezanejad et al., 2022; Ma et al., 2022; Wang Y. et al., 2019; Chen et al., 2019; Wan et al., 2021; Huang et al., 2024). Most of these models, however, suffer from a significant drawback: they are typically too complex in terms of parameters and require a large number of mathematical operations, making them unsuitable for industrial use or deployment on lightweight compute devices. Specifically, many of them need to calculate graphs of connectivity on top of point clouds multiple times, resulting in a large number of Floating Point Operations (FLOPs).

**Figure 1:**
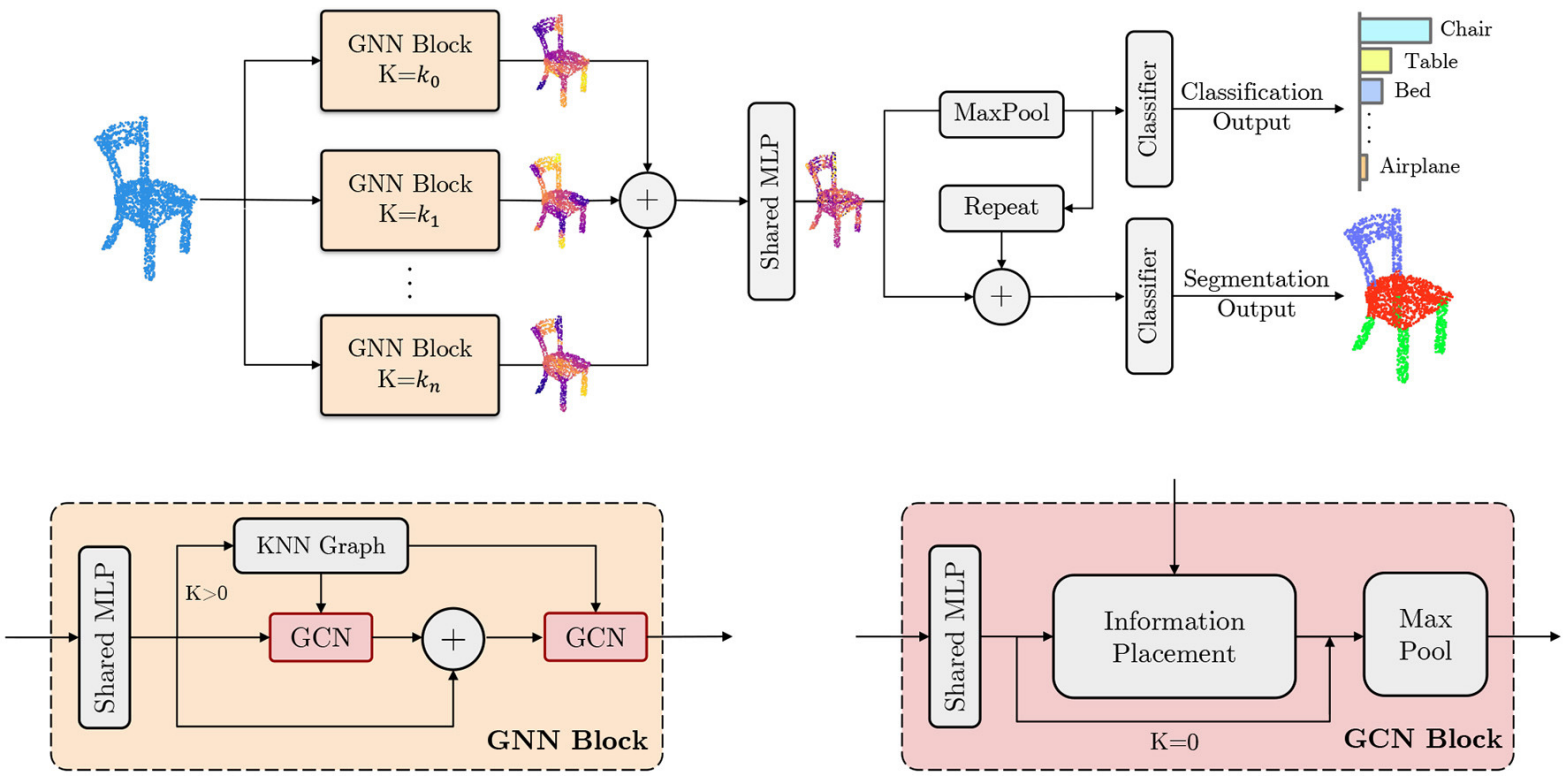
Overview of the MLGCN architecture. **(Top)** The complete architecture of the MLGCN model, designed for efficient 3D point cloud processing. The model consists of multiple branches of Graph Neural Network (GNN) blocks. Input points sampled from a 3D object are processed through these GNN blocks, which compute features at various spatial locality levels. These features are then used for downstream tasks such as object classification and part segmentation. **(Bottom)** Detailed structure of the GNN and Graph Convolutional Network (GCN) blocks, which serve as the fundamental components of the MLGCN model. The “+” symbol indicates the concatenation operation, allowing for flexible scaling by adding more GCN blocks as needed to enhance feature extraction and model performance.

The Multi-level Graph Convolution Neural Network (MLGCN) model is an ultra-efficient approach for 3D point cloud analysis that utilizes shallow Graph Neural Networks (GNN) blocks to extract features at various spatial locality levels. It employs precomputed KNN graphs shared among GCN blocks within a GNN block, significantly reducing computational overhead and memory usage. The model demonstrates competitive performance in object classification and part-segmentation tasks, achieving results comparable to state-of-the-art models while requiring up to a thousand times fewer floating-point operations and significantly less storage. Our work addresses the above limitations by introducing a lightweight model that can be trained easily and deployed on low-memory and low-end CPU devices. Instead of relying on complex and deep structures, such as attention mechanisms or deep stacks of feature extraction blocks, which require a large amount of training data and are susceptible to over-fitting, our proposed model (see Figure 1) consists of multiple shallow graph-based network blocks that capture information from point clouds using different graph KNNs. The k-Nearest Neighbors (KNN) algorithm is a simple, non-parametric method used for classification and regression, which predicts the label of a data point based on the majority class or average of its *k* closest neighbors in the feature space (Fix and Hodges, 1989). The use of different KNN graphs combined with shallow GNNs can alleviate the over-smoothing issue caused by deep GCNs (Li Q. et al., 2018; Zhou et al., 2020). Furthermore, utilizing precomputed shared graph KNNs within a GNN block greatly reduces the number of floating point operations. This architecture offers an efficient solution for processing point clouds without compromising accuracy, making it practical for real-world applications. The main contributions of this paper are as follows:

1. We propose a novel multi-branch, graph-based network that effectively captures features at various spatial locality levels of 3D objects using lightweight, shallow Graph Neural Network (GNN) blocks.
2. We demonstrate that our MLGCN model is significantly more efficient in terms of both computation and storage compared to existing approaches, without compromising on accuracy for downstream computer vision tasks.
3. We provide a thorough theoretical and experimental analysis of our model's performance, including the impact of each layer and component on the overall efficiency and accuracy.
4. We conduct extensive ablation studies to investigate the role and contribution of different branches within the model, offering deeper insights into its structure and functionality.

## 2 Related work

Over the past few years, the field of deep learning has seen a surge in research efforts aimed at developing effective methods for analyzing 3D sensor data. Methods that are designed for 2D images cannot be directly applied to 3D point clouds, which can be sparse, nonuniform in density, and lack local spatial ordering. A promising neural network model for 3D shape analysis in this setting is the PointNet model (Qi et al., 2017a). Unlike previous methods that transform point cloud data to regular 3D voxel grids or collections of images, PointNet processes point cloud data directly, extracting information from individual points and aggregating it into a feature vector using Global Max Pooling. The PointNet model's inability to capture local structures induced by the metric space limits its ability to represent fine-grained patterns and also generalize to complex scenes. To address this issue, PointNet++ (Qi et al., 2017b) applies PointNet recursively on nested partitions to extract local features, then combines the learned features across multiple scales.

In Qiu et al. (2021), the Geometric Back-projection Network (GBNet) combines channel affinity modules and CNN networks to improve the representation of point clouds, while learning both local and global features. GBNet utilizes an error-correcting feedback structure to design a back-projection CNN module. In Rezanejad et al. (2022), medial spectral coordinates are added as additional features to point cloud 3D coordinates. These coordinates contain both local and global features, resulting in improved performance of vanilla models for computer vision tasks. The PointMLP model (Ma et al., 2022) utilizes Multi-layer Perceptrons (MLP) to gather local information from points in a hierarchical manner without using a local feature extractor. Additionally, it employs lightweight affine modules to transform information from points to a normal distribution.

GNNs have the unique ability to handle topologically-structured data without requiring explicit encoding into vectors, by capturing graph-based information (Scarselli et al., 2009), making them an ideal candidate for the efficient processing of point clouds. The authors of Wang Y. et al. (2019) proposed the Dynamic Graph CNN (DGCNN) model where an EdgeConv neural network module incorporates local information around each point, and is then stacked to learn global shape properties using Graph Convolutional Networks. Han et al. (2024) proposed Mamba3D, a state space model with local geometric features designed specifically for point cloud learning. The model incorporates a Local Norm Pooling (LNP) block, which enhances local geometry extraction. Additionally, the proposed C-SSM feature reverse SSM alleviates pseudo-order reliance in unordered points. Zhang et al. (2019) enhanced DGCNN by introducing Linked Dynamic Graph CNN (LDGCNN), which links hierarchical features from different dynyamic graphs to calculate informative edge vectors. They removed the transformation network from DGCNN and showed that an MLP can extract transformation-invariant features. They further improved performance by freezing the feature extractor and retraining the classifier.

As attention mechanisms gained momentum in capturing node representation on graph-based data, Chen et al. (2019) proposed the Graph Attention-based Point Neural Network (GAPNet) model which embeds a graph attention mechanism within stacked MLP layers to learn local geometric representations. The GAP-Layer employs an attention-based graph neural network to consider the importance of each neighbor of a point. The Dilated Graph Attention-Based Network (DGANET) model (Wan et al., 2021) uses an improved KNN search algorithm to construct a local dilated graph for each point, modeling long-range geometric correlations with its neighbors. This helps the point neural network to learn more local features of each point, with a larger receptive field during the convolution operation. The authors embed an offset-attention mechanism into a dilated graph attention module and employ graph attention pooling to aggregate the most significant features. Huang et al. (2024) propose the Dual-Graph Attention Convolution Network (DGACN), which introduces an improved version of graph attention that leverages information from different hops. They also propose a novel graph self-attention mechanism that extracts more informative features from point clouds.

Li et al. (2021) proposed sharing KNN graphs among multiple GCNs to avoid redundant KNN calculations for each GCN. Additionally, the number of neighbors (*K*) in the GCNs increases as information progresses through the layers, ensuring a growing receptive field. This method also applies MLP before aggregating the feature. Although they manage to make DGCNN about five times faster, and about two times smaller, their solution is still relying on deep GNN blocks and is still very heavy as DGCNN is an extremely heavy model itself. The Point-transformer (Zhao et al., 2021) model utilizes self-attention to capture local information in the vicinity of each point. In addition, the authors introduce a trainable positional encoding that is learned within the end-to-end network. Wan et al. (2021), Huang et al. (2024), Wang L. et al. (2019), and Zhao et al. (2021) use graph attention-based mechanisms that are known to be parameter-heavy and can make the process of training and inference computationally expensive. Point-BERT (Yu et al., 2022) introduces a novel approach to adapting the Transformer architecture, popularized by BERT, for the task of processing 3D point clouds. Unlike traditional methods, the authors propose a Masked Point Modeling (MPM) pre-training task that involves dividing the point cloud into local patches and training Transformers to predict masked-out patches, enhancing the model's ability to understand complex 3D data. PointGPT (Chen et al., 2023) introduces a pre-training task focused on point cloud auto-regressive generation. It divides the input point cloud into structured patches, arranging them sequentially by spatial proximity. Leveraging an extractor-generator transformer decoder with dual masking, PointGPT learns latent representations from previous patches to predict the next point in an auto-regressive manner. Point2vec (Zeid et al., 2023) introduces a novel self-supervised representation learning approach that overcomes the shortcomings of data2vec (Baevski et al., 2022), enabling the acquisition of robust and transferable features through self-supervised learning.

## 3 The proposed method: MLGCN

MLGCN is a multi-level graph neural network model that can capture information from 3D point clouds at different locality levels efficiently. The model consists of multiple GNN blocks, each taking a set of 3D point clouds as input and learning a representation of the 3D dataset. The model then concatenates and uses these features for downstream tasks. We have designated two downstream branches: one for the classification task (i.e., correctly labeling the 3D model) and one for the segmentation task (i.e., decomposing the model into a set of semantically meaningful parts). In this section, we describe the key components of the MLGCN model, a schematic of which is shown in Figure 1. We assume the following point cloud as input to the system:


(1) $$\begin{array}{l} X=\left\{{\text{p}}_{i}=\left(x_{i},y_{i},z_{i}\right)\in {\mathbb{R}}^{3}\text{for}i=1,2,\cdots \;,N\right\} \end{array}$$

### 3.1 KNN graphs

Given 3D point cloud data, the model forms a set of KNN graphs, where the nodes represent 3D points, and each node is connected to its *k* closest nodes using edges. The parameter *k* defines the locality level around each point where local neighborhood information will be collected. The unique utilization structure of our KNN graph is that the graph is computed once for an input X and then reused for various other blocks' outputs of *Y*. This approach saves computation time and resources, making our model very efficient. The edge connectivity from the KNN graph is used to decide on passing information (messages) over an edge, allowing the model to capture the global features of the input data. Overall, the KNN graph used in our MLGCN model provides a way to explore the local structure of 3D point clouds as well as capture global features efficiently and effectively. To formulate the KNN graph construction, we define the graph $G_{k}$ as:


(2) $$\begin{array}{l} G_{k}=\left(X,E_{k}\right) \end{array}$$

where X represent the nodes in our graph and $E_{k}\subseteq X\times X$ represents the edges. Each node **p**_*i*_ is connected to another node **p**_*j*_ if **p**_*j*_ locates within the *k* closest neighbors of **p**_*i*_. As the graph is directed, the graph contains self-loops (see Figure 1 bottom left).

### 3.2 GNN block

Each Graph Neural Network (GNN) block takes a 3D point cloud as input and extracts features from it. These features are then concatenated and used for both classification and segmentation tasks. To extract these features, the GNN block applies a series of operations on the input data. First, a multi-layer perceptron (MLP) is applied to transform the input, which is then processed by a series of Graph Convolution Network (GCN) blocks and one single KNN graph. If the parameter *k* is set to 0, the model skips the KNN graph computation and extracts only global information from the point cloud.

Each GCN block processes the input data and then its output is concatenated with its input and passed to the next GCN block, along with the output of the KNN graph. The KNN graph output is shared among GCN blocks within a GNN block. The next GCN block operates similarly to the previous one, processing the concatenated features to extract additional information. This process can be repeated multiple times except for the last GCN block where the input and output vectors are no longer concatenated. In Figure 1 bottom left, we illustrate the GNN block architecture. Here


(3) $$\begin{array}{l} \Gamma \left(X\right)=f\left(\text{concat}\left\{GB_{k_{i}}\left(X\right)|i=1,\cdots \;,m\right\}\right), \end{array}$$

where *f* is the shared MLP applied to the concatenated outputs of the GNN blocks GB(X).

### 3.3 GCN block

The GCN block in our MLGCN model applies a series of operations on the input data using the KNN graph information that was computed previously. The input data is first processed by a shared multi-layer perceptron. The GCN block then uses the KNN graph information to propagate the input feature information for each node and the nodes it is connected to. This operation allows the model to capture local features of the input data using the precomputed KNN graph. The output of the GCN block is then max pooled. This max pooling operation summarizes the information learned from the input data and allows the model to capture the most important features of the input with respect to the defined locality level *k*.

Our information placement module uses graph connectivity as follows. We assume our message passing function *h*(**p**_*i*_, **p**_*j*_, *Y*) accepts two nodes **p**_*i*_, **p**_*j*_ and then passes the information (*y*_*j*_∈*Y*) on node **p**_*j*_ to node **p**_*i*_ conditioned on the graph neighborhood information, i.e., if (**p**_*i*_, **p**_*j*_)∈*E*_*k*_. Here *E*_*k*_ is shared among all GCN blocks that belong to the same GNN block. In Figure 1 bottom right, we show the GCN block architecture.

### 3.4 Information processing in the GCN block

As mentioned previously, a GNN block input is a 3D point cloud X where a graph $G_{k}=\left(X,E_{k}\right)$ is made. We now explain how the inputs and outputs of each GCN block are obtained. Let $y_{i}^{t}$ represent the information from the *i*^*th*^ node of our graph after the *t*^*th*^ GCN block operation is applied on the input. We can formulate $y_{i}^{t}$ as


(4) $$\begin{array}{l} y_{i}^{t}=A\left(\left\{h\left({\text{p}}_{i},{\text{p}}_{j},f_{t}\left(Y^{*\left(t-1\right)}\right)\right)|\left({\text{p}}_{i},{\text{p}}_{j}\right)\in E_{k}\right\}\right) \end{array}$$

where *A* is the aggregation function and *f*_*t*_ is the *t*th shared MLP. The aggregation function used in our pipeline is max pooling (although other aggregation functions could be used as well) and it is applied along the neighborhood axes. For all GCN blocks except for the last one, the information is $y_{i}^{t}$ concatenated with the input to the same GCN block:


(5) $$\begin{array}{l} y_{i}^{*t}=\text{concat}\left(y_{i}^{t},y_{i}^{*\left(t-1\right)}\right). \end{array}$$

For *t* = 1, $y_{i}^{t}=y_{i}^{*t}=f_{0}\left(X\right)$. Now, with the GNN block represented by GB(X), $GB\left(X\right)=Y^{l}$ where *l* is the index of the last GCN block.

### 3.5 Overall architecture

Each variation of MLGCN uses a set of GNN blocks with different values of *k*. Let the first block be a block with *k* = 0, with the purpose of extracting global information for each node. The other blocks can be set to extract information with different locality levels. Now assume we have a set of *m* different GNN blocks in our model with *K* = {*k*_1_, *k*_2_, ⋯ , *k*_*m*_}. As mentioned previously, outputs of all GNN blocks are concatenated and then passed through a shared MLP. From there, the extracted features are pooled and then used in a downstream task, e.g., classification or segmentation.

#### 3.5.1 Classification branch

We designated a classification branch to classify 3D input models according to different labels. For the classification task, we simply apply a max pooling along the node's axes and pass the outcome to a classifier as follows:


(6) $$\begin{array}{l} L_{\text{classification}}=C\left(A\left(\Gamma \left(X\right)\right)\right) \end{array}$$

where $L_{\text{classification}}$ is the set of classification labels, C is a classifier and *A* is the max pooling function here.

#### 3.5.2 Segmentation branch

The second designated branch in our overall architecture is dedicated to the part segmentation of the 3D models. For the segmentation task, the model concatenates the information of each node with the repeated pooled information obtained for all the nodes from the GNN blocks that is used in the classification branch:


(7) $$\begin{array}{l} L_{\text{segmentation}}=C\left(\text{concat}\left(\text{repeat}\left(\Gamma \left(X\right)\right),\Gamma \left(X\right)\right)\right) \end{array}$$

where $L_{\text{segmentation}}$ is the set of segmentation labels, C is a classifier and *A* is the max pooling function.

### 3.6 Light-MLGCN and Lighter-MLGCN

Here, we introduce two sample architectures with an MLGCN backbone, Light-MLGCN, and Lighter-MLGCN. These are example models to demonstrate the efficiency of MLGCN-based models. To show this, we compare their performance to that of state-of-the-art models that are commonly used for 3D classification and segmentation problems.

Both Light-MLGCN and Lighter-MLGCN utilize multiple GNN-blocks with varying *k* sizes. This allows them to capture information related to different locality levels without requiring additional trainable parameters to capture the distance from the neighborhood center. Additionally, the *l* value for each GNN block is set to 2, resulting in a shallow network that is less susceptible to over-fitting. Moreover, Light-MLGCN computes graphs based on only three features, as the range of *f*_0_ is 3, which makes its graph calculation process much faster than that of other existing papers. These models share the graph for each GNN-block, which results in fewer mathematical operations. Light-MLGCN was trained using hyperparameters of *K* = 63, 15, 0, and for each GNN block, *y*^0^∈ℝ^1024 × 3^, *y*^1^∈ℝ^1024 × 32^, *y*^2^∈ℝ^1024 × 128^, and $\Gamma \left(X\right)\in {\mathbb{R}}^{1024\times 256}$. Conversely, Lighter-MLGCN was trained using hyperparameters of *K* = 31, 7, 0, and for each GNN block, *y*^0^∈ℝ^512 × 3^, *y*^1^∈ℝ^512 × 16^, *y*^2^∈ℝ^512 × 64^, and $\Gamma \left(X\right)\in {\mathbb{R}}^{512\times 128}$.

## 4 Experiments

We now evaluate the performance of our MLGCN models with respect to different metrics. We demonstrate that our models achieve comparable accuracy to existing models in both classification and segmentation tasks while being considerably smaller and faster.

### 4.1 Implementation details

We trained our models on a machine with a single P100 GPU with 12GB memory. For the optimization step, we employed the Adam optimizer, setting the batch size to 128. The initial learning rate was 0.001, which was reduced by a factor of 0.997 (*e*^−0.003^) after the 20th epoch.

### 4.2 Classification

Our primary experiment involves comparing the accuracy and speed of our models on ModelNet-40 (Wu et al., 2015), a dataset consisting of 9,843 training and 2,468 testing meshed CAD models from 40 distinct categories. In Table 1, we compare our model to several recent and popular models in terms of accuracy, floating-point operations, number of trainable parameters, model storage size, and GPU memory.

**Table 1:** We carry out a comparison of various models using different metrics, with processing on the ModelNet-40 dataset as the basis for evaluation.

**Method**	**Type**	**Input**	**Model size**	**FLOPS**	**Number of parameters**	**Accuracy**	**GPU memory**
		**Shape**	**Mega bytes**	**(100 mega)**	**100 thousands**		**Mega bytes**
Pointnet (vanilla)	Non-GNN	1,024	3.5	1.5	8	87.1	19
Pointnet			1,024	38	4.5	35	89.2	50
Pointnet++			1,024	17	8.9	14	90.7	100
GBNet			1,024	34	98	87	93.8	220
PointMLP		1,024	100	157	132	**94.5**	90
DGCNN	GNN	1,024	21	1,300	18	92.9	110
LDGCNN			1,024	13	920	10	92.9	–
DGANET			1,024	6	–	15	92.3	–
GAPNet			1,024	21	580	19	92.4	31
DGACN			1,024	–	1,600	240	94.1	–
Point-transformer			1,024	82	–	140	93.7	155
Light MLGCN	GNN	1,024	**1.5**	**1.3**	**1.2**	90.7	45
Lighter MLGCN			**512**	**0.4**	**0.2**	**0.3**	88.6	**10**

In every column except for the accuracy column, the lowest value is bold. In the accuracy column, the highest value is bold.

As shown in Table 1, when comparing Light-MLGCN with the best model in terms of accuracy, we see that it is more than 100 times more efficient in terms of FLOPS, and is also more than 100 times smaller in terms of the number of parameters and more than 60 times smaller in terms of model size. Whereas it has only 3.8% lower classification accuracy on the ModelNet-40 dataset than the best model (Wu et al., 2015), Light-MLGCN is considerably faster and more compact.

Among graph-based models, DGACN achieves the highest accuracy but requires 1,230 times more FLOPS than our model while only achieving 3.4% higher accuracy. Additionally, Lighter-MLGCN achieves comparable accuracy to Light-MLGCN with only a 2.1% difference, while being significantly smaller and faster and processing only 512 points sampled from point clouds. A detailed presentation of our results is in Table 1.

#### 4.2.1 Classification on ScanObject

ScanObject (Uy et al., 2019) is a more recent dataset than ModelNet-40, with 15,000 3D objects from 15 different classes. Unlike Modelnet-40, whose objects are synthetic, ScanObject's 3D models are real, and as a result, contain background noise and are sometimes partially occluded. To test the ability of our models, we tested Light-MLGCN and Lighter-MLGCN on PB_T50_RS, which is the hardest variant of the ScanObject dataset. The results are shown in Table 2.

**Table 2:** Accuracy and mean accuracy of models compared to LightMLGCN.

**Model**	**Accuracy**	**Mean accuracy**
3DmFV (Ben-Shabat et al., 2018)	63.0	58.1
PointNet (Qi et al., 2017a)	68.2	63.4
SpiderCNN (Xu et al., 2018)	73.7	69.8
PointNet++ (Qi et al., 2017b)	77.9	75.4
DGCNN (Wang Y. et al., 2019)	78.1	73.6
PointCNN (Li Y. et al., 2018)	78.5	75.1
BGA-PN++ (Uy et al., 2019)	80.2	77.5
BGA-DGCNN (Uy et al., 2019)	79.7	75.7
LightMLGCN	77.3	74.2
LighertMLGCN	74.1	70.8

### 4.3 Segmentation

In addition to the 3D classification problem, we also evaluated the performance of our models on the part segmentation task using the ShapeNetPart dataset (Yi et al., 2016). This dataset contains 16,881 3D shapes from 16 different classes, where each class has two–six parts, resulting in a total of 50 different parts. Our objective is to demonstrate that our lightweight model can achieve comparative results (or even better results) while remaining significantly smaller in size than other existing models. To ensure a fair comparison with previous work, we trained and tested our model on samples comprising 2,048 points each, using the same settings as those in other papers. The results are presented in Table 3, which shows that our model achieves comparable performance with other state-of-the-art models, despite being much smaller in size.

**Table 3:** A comparison of the results achieved by different models for part segmentation on the ShapeNetPart dataset.

**Method**	**Class**	**Inst**.	**Aero**	**Bag**	**Cap**	**Car**	**Chair**	**Ear-Phone**	**Guitar**	**Knife**	**Lamp**	**Laptop**	**Motor-Bike**	**Mug**	**Pistol**	**Rocket**	**Skate-Board**	**Table**
	**mIoU**	**mIoU**																
Pointnet	80.4	83.7	83.4	78.7	82.5	74.9	89.6	73.0	91.5	85.9	80.8	95.3	65.2	93.0	81.2	57.9	72.8	80.6
Pointnet++	81.9	85.1	82.4	79.0	87.7	77.3	90.8	71.8	91.0	85.9	83.7	95.3	71.6	94.1	81.3	58.7	76.4	82.6
GBNet	82.6	85.9	84.5	82.2	86.8	78.9	**91.1**	74.5	91.4	89.0	84.5	95.5	69.6	94.2	83.4	57.8	75.5	83.5
PointMLP	**84.6**	**86.1**	83.5	83.4	87.5	**80.5**	90.3	78.2	92.2	88.1	82.6	96.2	**77.5**	**95.8**	**85.4**	**64.6**	83.3	84.3
DGCNN	82.3	85.2	84.0	83.4	86.7	77.8	90.6	74.7	91.2	87.5	82.8	95.7	66.3	94.9	81.1	63.5	74.5	82.6
LDGCNN	82.2	84.8	84.0	83.0	84.9	78.4	90.6	74.4	91.0	88.1	83.4	95.8	67.4	94.9	82.3	59.2	76.0	81.9
DGANET	82.6	85	84.6	**85.7**	87.8	78.5	91.0	77.3	91.2	87.9	82.4	95.8	67.8	94.2	81.1	59.7	75.7	82.0
GAPNet	82	84.7	84.2	84.1	**88.8**	78.1	90.7	70.1	91.0	87.3	83.1	96.2	65.9	95.0	81.7	60.7	74.9	80.8
MLGCN	83.2	84.6	**87.4**	78.2	85.6	75.6	75.9	**81.1**	**93.1**	**93.2**	**89**	**96.4**	67.5	93.7	81.8	60.6	**85.2**	**87.6**

The results demonstrate that our proposed model performs comparably to the best-performing models in the literature for part segmentation, and in some cases, even outperforms them. We obtain the best score for eight out of 16 object classes. The highest value in each column is bold.

**Figure 2:**
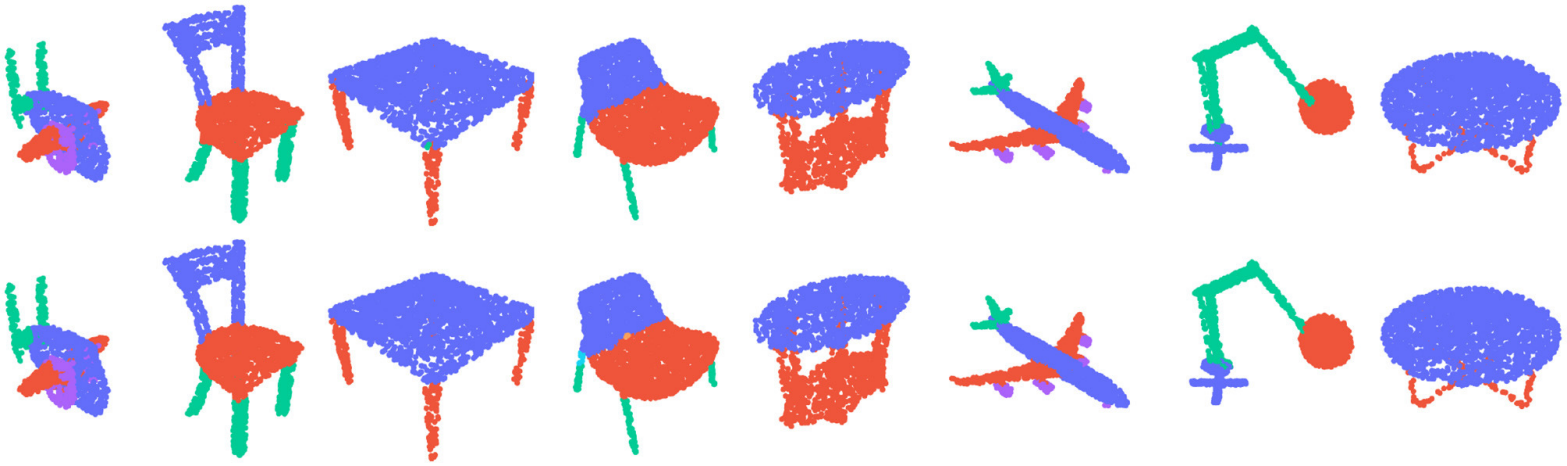
The **top row** shows the ground truth segmentation, while the **bottom row** displays the predicted class output label using our MLGCN model.

Moreover, to provide a visual representation of our model's output, we compared its output labels to the ground truth in Figure 2. The results show that our model is able to accurately segment the parts of the 3D objects, further demonstrating its efficacy for this task.

## 5 Ablation studies

We now examine details of our models and demonstrate that they are much more efficient than the other existing models.

### 5.1 FLOPS required for each operation

To support the idea of MLGCN, we study the effect of each layer in our models on the number of floating point operations. In Table 4, the complexity of each layer in LightMLGCN, in terms of floating-point operations, is shown. In many graph-based models, graph calculation is one of the most computationally intensive operations. To calculate the K-NN graph, the *K*-nearest neighbor algorithm is used to find the nearest neighbors of each point. This results in a computational complexity of *O*(*n*^2^×*f*), where *n* is the number of points and *f* is the length of the feature vector for each point. Note that there are other algorithms that are not brute-force and require less number of operations, but they all are not well parallelizable and therefore not even nearly as efficient as the brute-force method. This complexity can have a significant impact on the number of floating-point operations required for a graph-based model.

**Table 4:** Here are the complexity of each layer in LightMLGCN.

**Layer**	**FLOPS**
KNN	*O*(*n*^2^×*f*)
GCN	*O*(*n*×*f*×*k*)
Dense	*O*(*n*×*f*_1_×*f*_2_)

*n* is the number of points in a 3D Object, *f* is the number of features, *k* is the number of nearest neighbors, *f*_1_ and *f*_2_ are the number of in and out features in a Fully connected layer respectively.

Table 5 demonstrates that graph calculation can be highly resource-intensive when dealing with a large number of points and features. For instance, the FLOPS required to calculate graphs using KNN can increase dramatically as the number of points and features increase. In contrast, Light-MLGCN employs shared graphs on small feature vectors for multiple GCNs, resulting in reduced computational overhead. As a result, Light-MLGCN is able to achieve comparable performance to other state-of-the-art models while being much faster and smaller in size.

**Table 5:** We provide a comparison of the number of floating-point operations (FLOPS) required for different operation types in a model.

**Dimension**	**Operation type**	**FLOPS**
**Configuration**		**Mega**
(1,024.3)–(1,024.32)	Point-wise dense	0.13
(1,024.32)–(1,024.64)	Point-wise dense	2
(1,024.64)–(1,024.128)	Point-wise dense	8
(1,024.128)–(1,024.256)	Point-wise dense	33
(1,024.512)–(1,024.1024)	Point-wise dense	537
(2,048.128)–(2,048.256)	Point-wise dense	67
(2,048.512)–(2,048.1024)	Point-wise dense	1,074
(1024.3)	Graph calculation	4
(1,024,32)	Graph calculation	50
(1,024.64)	Graph calculation	100
(1,024.128)	Graph calculation	201
(1,024.512)	Graph calculation	805
(2,048.128)	Graph calculation	805
(2,048.512)	Graph Calculation	3,221

Most current graph-based models used for this specific problem require multiple instances of graph extraction on point clouds with 32–128 features. This can result in a large number of floating-point operations, which can lead to reduced performance and longer training times. As shown in Table 1, graph-based models generally require significantly more floating-point operations than non-graph-based models.

### 5.2 Performance of MLGCN model with various input shapes

While Light-MLGCN was primarily designed to operate on 1,024 points and Lighter-MLGCN on 512 points, both models can be tested on other sampled point cloud sizes. This section aims to demonstrate the effectiveness of our models with different point cloud shapes. We show that our models can perform well even on sparser point clouds. To get a better sense of this, we tested both of our models with input sizes of 128, 256, 512, and 1,024 and present the number of FLOPS and their corresponding accuracies in Table 6.

**Table 6:** The performance of the MLGCN model can vary with different input shapes.

**Model**	**Input shape**	**FLOPS (Giga)**	**Accuracy**
Light	1,024	0.13	90.7
		512	0.06	89.5
		256	0.03	88.4
		128	0.014	86.4
Lighter	1,024	0.04	89.8
		512	0.017	88.6
		256	0.008	86.9
		128	0.004	83.7

In order to evaluate the robustness of the model under different input conditions, we conducted experiments with various input shapes and analyzed the results.

As shown in this table, the simplicity and shallow structure of both Light-MLGCN and Lighter-MLGCN enable them to be trained on smaller point cloud samples without over-fitting, resulting in high accuracy even when using much fewer 3D point cloud sample points. This demonstrates the flexibility of our models and their ability to perform well under varying input conditions.

### 5.3 MLGCN as an encoder

**Figure 3:**
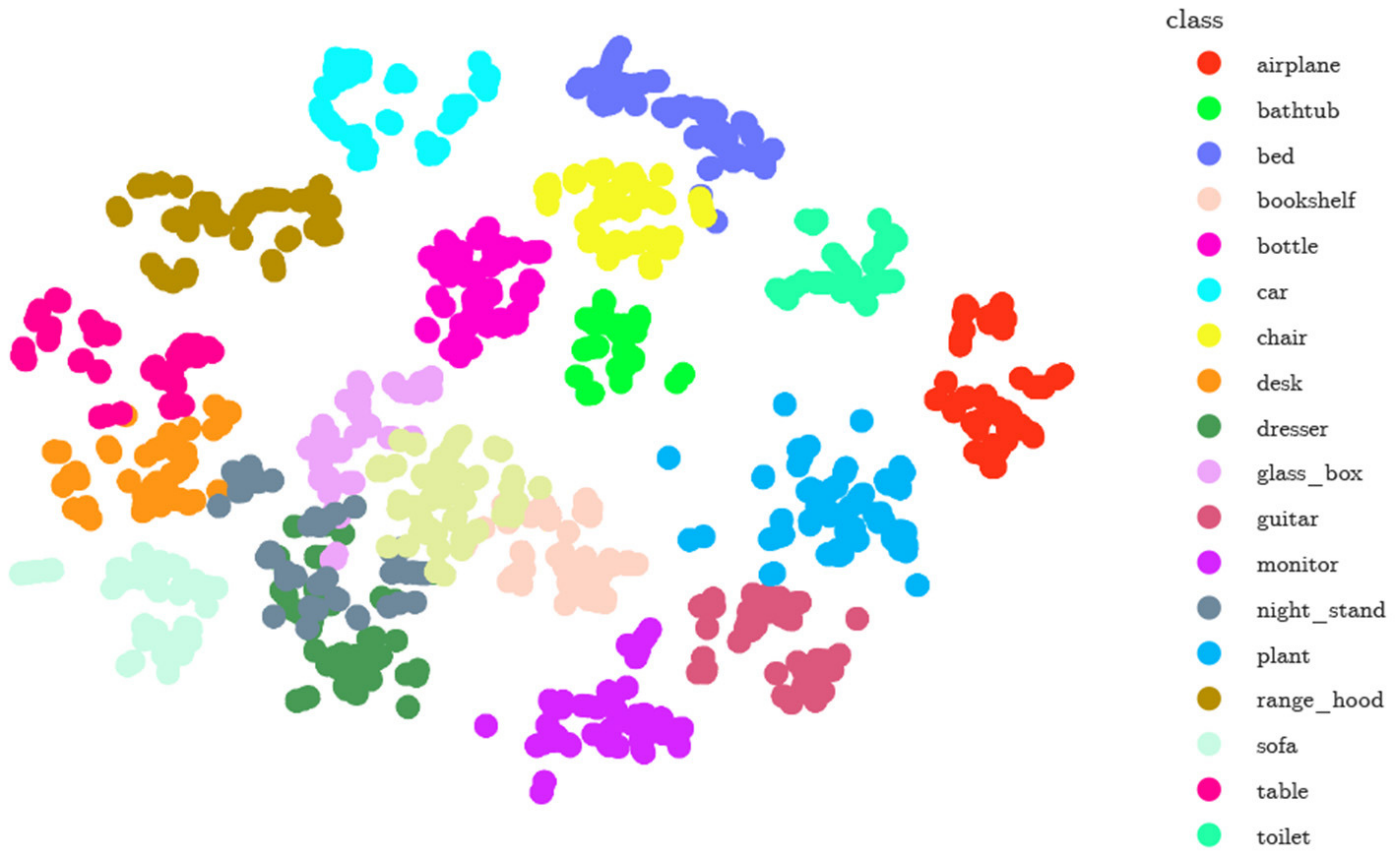
A 2D TSNE plot to visualize the projected features obtained by our proposed model (Light-MLGCN) for 20 different object classes.

Our proposed MLGCN model can also serve as an encoder model for encoding 3D point clouds and extracting meaningful features. To evaluate this hypothesis, we extracted the information of the classification MaxPool(Γ(X)) branch (before the classifier) and projected it into a lower-dimensional space to examine how these features separate between different classes of 3D models. Figure 3 presents a (2D TSNE) visualization of the projection of feature vectors generated by our model when tested on the Modelnet-40 dataset onto a 2-dimensional space. The figure clearly demonstrates that our model can effectively cluster each class of 3D objects into a separate cluster, indicating the ability of the model to extract and encode meaningful features from 3D point clouds. It should be noted that *Z*-score outlier detection was applied to the data. The figure suggests that our proposed model can serve as a robust encoder model for extracting features from 3D point clouds.

## 6 Role of different sets of *K*

In this section, we examine the influence of different *K* values in the GNN blocks on the performance of the MLGCN model. The parameter *K* controls the number of nearest neighbors used in constructing the KNN graph, thereby affecting the scale at which features are extracted from the point cloud.

### 6.1 Impact of different *K* combinations

As shown in Table 7, we conducted experiments using various sets of *K* values, including our baseline configuration of ({0, 15, 63}), where *K* = 0 captures global features, and *K* = 15 and *K* = 63 capture increasingly broader local features. This configuration provided the best trade-off between accuracy and computational efficiency on the ModelNet-40 dataset. We also tested alternative sets such as ({0, 19, 63}) and ({0, 9, 44}). While these combinations performed competitively, the baseline ({0, 15, 63}) set consistently achieved the highest accuracy, balancing the capture of both local and global features.

**Table 7:** The outcomes of our proposed model when using different sets of *K* in the GNN blocks.

**Block**	**FLOPS (Giga)**	**Accuracy**
[0, 15, 63]	0.13	90.7
[0, 19, 63]	0.13	90.4
[0, 9, 44]	0.13	90.3
[0, 19, 44]	0.13	90.1
[15, 63]	0.09	90.1
[0, 44]	0.08	89.9
[19, 63]	0.09	89.9
[9, 44]	0.09	89.8
[15]	0.04	89.5
[44]	0.05	89.3
[63]	0.05	89.1

### 6.2 Importance of global features

Our findings highlight the significance of including *K* = 0 to capture global features. Incorporating global information consistently enhances model accuracy, as it provides a broad context that complements the detailed local features extracted by higher *K* values.

### 6.3 Multi-scale information capture

By combining different *K* values, the MLGCN model effectively captures multi-scale information, where each GNN block focuses on a specific locality level. This multi-scale approach ensures that both local details and global patterns are well-represented, improving the model's robustness in classification and segmentation tasks.

### 6.4 Efficiency considerations

The choice of *K* values also affects the computational cost. Larger *K* values (e.g., *K* = 63) capture more extensive local structures but require more computations. It is crucial to balance capturing sufficient locality with maintaining efficiency, particularly for real-time applications. In summary, the selection of *K* values is crucial for optimizing both the performance and efficiency of the MLGCN model. Adjusting *K* allows the model to be tailored to specific tasks, ensuring a balance between accuracy and computational demands.

## 7 Conclusion

In this paper, we introduced the Multi-level Graph Convolutional Neural Network (MLGCN) model, designed as a lightweight and efficient solution for 3D shape analysis, particularly for tasks such as 3D object classification and part segmentation from point cloud data. Our approach is driven by the need for models that balance high accuracy with low computational and memory demands, making them suitable for practical deployment in industrial and mobile applications.

The MLGCN model achieves this balance by leveraging shallow Graph Neural Network (GNN) blocks, which utilize precomputed KNN graphs shared across these blocks to extract features from 3D point clouds at various spatial locality levels. This design significantly reduces the computational overhead and memory usage typically associated with 3D deep learning models, while still maintaining competitive performance.

Our extensive experiments demonstrate that the MLGCN model outperforms many state-of-the-art models in terms of efficiency, requiring far fewer operations and parameters while still achieving high accuracy in both classification and segmentation tasks. Additionally, we explored the impact of various parameters and model configurations, providing insights into the trade-offs between model complexity and performance.

Despite these advances, the choice of K values within the GNN blocks remains an area with potential for further exploration. Future work will aim to refine these parameters and extend the application of MLGCN to real-world scenarios, such as real-time LiDAR data processing, to evaluate the model's effectiveness in more complex environments.

Overall, MLGCN represents a significant step forward in the development of efficient and scalable 3D shape analysis models, with broad implications for applications in robotics, augmented reality, and beyond. We hope that this work will inspire further research into creating even more efficient and lightweight models for 3D vision tasks in the future.

## Data Availability

The original contributions presented in the study are included in the article/supplementary material, further inquiries can be directed to the corresponding author.
